# Image quality and acquisition time assessments for phase oversampling in compressed sensing sensitivity encoding: Comparison with conventional SENSE

**DOI:** 10.1002/acm2.13509

**Published:** 2021-12-24

**Authors:** Ji Sung Jang, Ho Beom Lee, Chong Hyun Suh, Min Hee Lee

**Affiliations:** ^1^ Departments of Radiology and Research Institute of Radiology Asan Medical Center College of Medicine University of Ulsan South Korea

**Keywords:** compressed sensitivity encoding, image quality, phase oversampling, sensitivity encoding

## Abstract

This study compared sensitivity encoding (SENSE) and compressed sensing sensitivity encoding (CS‐SENSE) for phase oversampling distance and assessed its impact on image quality and image acquisition time. The experiment was performed with a large diameter phantom using 16‐channel anterior body coils. All imaging data were divided into three groups according to the parallel imaging technique and oversampling distances: groups A (SENSE with phase oversampling distance of 150 mm), B (CS‐SENSE with phase oversampling distance of 100 mm), and C (CS‐SENSE with phase oversampling distance of 75 mm). No statistically significant differences were observed among groups A, B, and C regarding both T2 and T1 turbo spin‐echo (TSE) sequences using an acceleration factor (AF) of 2 (*p* = 0.301 and 0.289, respectively). In comparison with AF 2 of group A, the scan time of AF 2 of groups B and C was reduced by 11.2% and 23.5% (T2 TSE) and 15.8% and 22.7% (T1 TSE), respectively, while providing comparable image quality. Significant image noise and aliasing artifact were more evident at AF ≥ 2 in group A compared with groups B and C. CS‐SENSE with a less phase oversampling distance can reduce image acquisition time without image quality degradation compared with that of SENSE, despite the increase in aliasing artifact as the AF increased in both CS‐SENSE and SENSE.

## 1 INTRODUCTION

Several different parallel imaging techniques were introduced to reduce data acquisition time in magnetic resonance imaging (MRI).^1^, ^2^, ^3^ In recent years, compressed sensing (CS) and hybrid technique (CS‐SENSE), for example, combination of CS and sensitivity encoding (SENSE), were widely used in clinical practice.

The image‐based SENSE technique theoretically does not require an extra field of view (FOV) given appropriate coverage. While those scans with prescribed FOV smaller than the target anatomy do require extra FOV called oversampling distance in the phase encoding direction with increasing acceleration factor (AF), which is defined as the ratio between a fully‐sampled data and an under‐sampled data.^1^, ^4^ As the spacing distance between *k*‐space lines is inversely proportional to the FOV, the increase in AF results in a reduced FOV image from each of the coil elements. Thus, the Nyquist sampling theorem criterion is not met, appearing with aliasing artifacts in the reduced FOV.^5^ To overcome this problem, CS‐SENSE consists of a variable density incoherent undersampling scheme that optimizes the balance between random basis and SENSE sampling using iterative reconstruction. Oversampling distance in the phase encoding direction is related to the data acquisition time due to the increase in the number of phase encoding steps, causing longer scan time. Hence, it is important to properly adjust phase oversampling distance and shorten the image acquisition time to avoid aliasing artifacts while using the AF. However, it is believed that most SENSE or CS‐SENSE studies have not mentioned phase oversampling distance to acquire images without aliasing artifact.^6^, ^7^, ^8^, ^9^ Moreover, no study focusing on the comparison of phase oversampling distance between SENSE and CS‐SENSE exists.

Therefore, this study aims to compare SENSE and CS‐SENSE for phase oversampling distance and assess its impact on image quality and acquisition time.

## 2 MATERIALS AND METHODS

### 2.1 Phantom and study design

This study used a large diameter phantom (Philips Healthcare, Eindhoven, The Netherlands) with a diameter of 40 cm and 45 small circular holes, each separated by 5.0 cm for the experiments. The phantom was filled with copper sulfate that enables the holes in the phantom to be shown as hyperintense objects in the MR image, thereby evaluating them for image distortion. The phantom was carefully positioned and aligned by fixing the support device. All imaging data were divided into three groups according to the parallel imaging technique and oversampling distances: groups A (SENSE with phase oversampling distance of 150 mm), B (CS‐SENSE with phase oversampling distance of 100 mm), and C (CS‐SENSE with phase oversampling distance of 75 mm).

### 2.2 MR equipment and scan parameters

All images were scanned on a clinical 70‐cm bore 3.0T MRI scanner with 45 mT/m maximum gradient strength and 200 T/m/s maximum slew rate (Ingenia; Philips Healthcare). In addition, 16‐channel anterior body coils (Philips Healthcare) were used for image acquisitions. The turbo spin‐echo (TSE) pulse sequence was used to acquire T1‐ and T2‐weighted imaging in the coronal orientation with the scan parameters FOV (300 × 300 mm), voxel size (1.2 × 1.2 mm), acquisition matrix (256 × 256), reconstruction matrix (512 × 512), number of excitations (1), slice thickness/slice gap (4/0 mm), number of slices (30), TSE factor of T2 (16), TSE factor of T1 (4), SENSE AFs (1.5, 2, 3, and 4), CS‐SENSE AFs (1.5, 2, 3, and 4), and phase encoding direction (head to feet). A further detailed summary of the parameters is presented in Table 1.

**TABLE 1:** Summary of detailed image acquisition parameters

**T2 TSE**				
Group A (using a SENSE with phase oversampling distance of 150 mm)				
Acceleration factor	1.5	2	3	4
TR (ms)	3016	3016	3016	3016
TE (ms)	65	65	65	65
Bandwidth (Hz)	217.6	217.6	217.6	217.6
Scan time (s)	121	89	63	47
Group B (using a CS‐SENSE with phase oversampling distance of 100 mm)				
Scan time (s)	100	79	53	42
Group C (using a CS‐SENSE with phase oversampling distance of 75 mm)				
Scan time (s)	89	68	47	37

T1 TSE				
Group A (using a SENSE with phase oversampling distance of 150 mm)				
Acceleration factor	1.5	2	3	4
TR (ms)	594	594	594	594
TE (ms)	14	14	14	14
Bandwidth (Hz)	688.9	688.9	688.9	688.9
Scan time (s)	134	101	68	53
Group B (using a CS‐SENSE with phase oversampling distance of 100 mm)				
Scan time (s)	113	85	59	45
Group C (using a CS‐SENSE with phase oversampling distance of 75 mm)				
Scan time (s)	101	78	53	40

*Notes*: AF, acceleration factor; TE, echo time; TR, repetition time; TSE, turbo spin‐echo. All image acquisition parameters are the same for all three groups except for oversampling distance and scan time.

### 2.3 Image analysis

**FIGURE 1:**
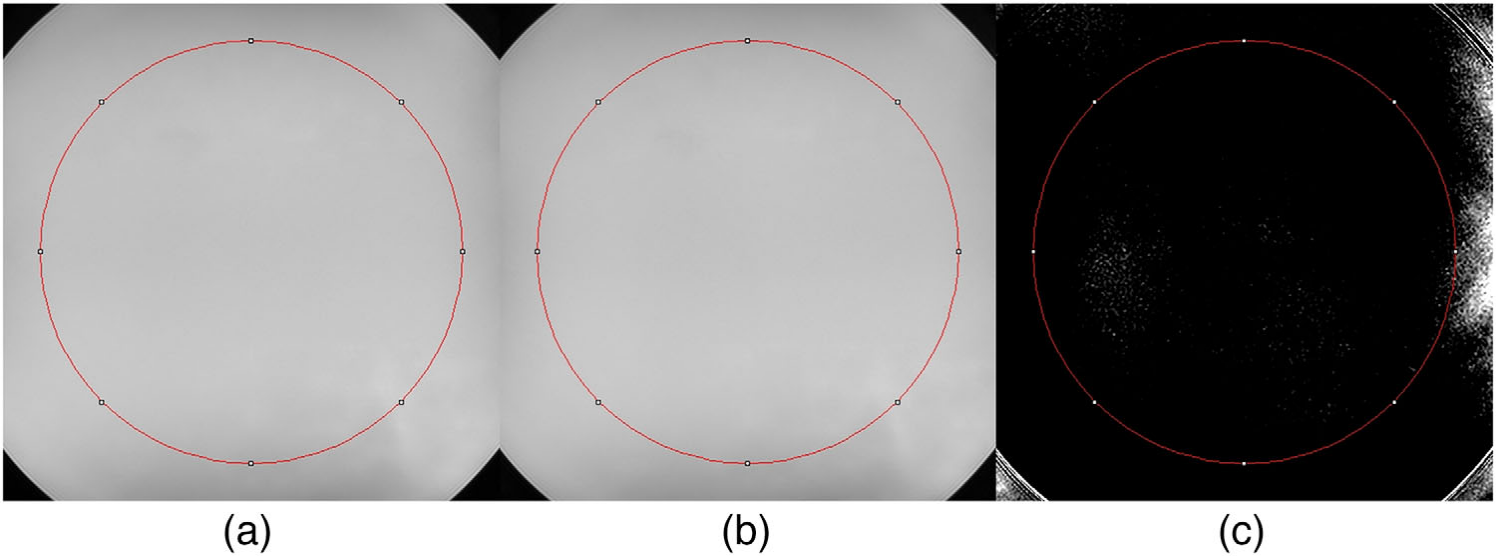
Region of interest placement for measuring signal‐to‐noise ratio. (a) First image acquired with the same imaging parameters, (b) second image acquired with the same imaging parameters, and (c) third image subtracted from the first and second images to acquire a standard deviation of the subtraction image

The structural similarity index (SSIM) tool was analyzed as an image quality assessment using MATLAB (R2016b; MathWorks, Natick, MA, USA). This SSIM was calculated using the equation

(1) $$\begin{array}{ccc} & & \mathrm{SSIM}\left(x,y\right)=\left(2\mu_{x}\mu_{y}\;+\;C_{1}\right)\left(2\sigma_{xy}\;+\;C_{2}\right)\\ & & /\left({\mu_{x}}^{2}\;+\;{\mu_{y}}^{2}\;+\;C_{1}\right)\left({\sigma_{x}}^{2}\;+\;{\sigma_{y}}^{2}\;+\;C_{2}\right), \end{array}$$

where μ*_x_*, μ*_y_*, σ*_x_*, σ*_y_*, and σ*_xy_* are the local means, standard deviations, and cross‐covariances for images *x* and *y*, respectively. This value indicated from 0 to 1 and was ∼1 when the two images were nearly identical.^10^, ^11^ Moreover, the signal‐to‐noise ratio (SNR) was calculated using the National Electrical Manufacturers Association subtraction method according to the equation:^12^, ^13^

(2) $$\mathrm{SNR}\;=\sqrt{2}\;\frac{\mathrm{Mean}\;\mathrm{signal}\;\mathrm{value}}{\sigma },$$

where the mean signal value of the two images for subtraction and σ is the standard deviation of the subtracted images, which is related to two images obtained from identical parameters (a subtraction image was performed to make a noise‐only image). The mean signal value and σ were acquired from the corresponding 85% region of interest in the two images and the subtracted image, respectively (Figure 1). The $\sqrt{2}$ value is required because noise with a propagation of error is obtained from the difference image.^14^ The SNR analysis was performed using Image J (Bethesda, MD, USA; http://rsbweb.nih.gov/ij/).

### 2.4 Statistical analysis

The Kolmogorov–Smirnov test was used to confirm the SNR and SSIM values following a normal distribution. All values among the three groups were compared using the analysis of variance based on the results of the Kolmogorov–Smirnov test. Moreover, post hoc tests were performed using the Tukey–Kramer method when statistically significant differences were indicated. Statistical analyses were performed using IBM SPSS Statistics for Windows/Macintosh, v. 21.0 (IBM Corp., Armonk, NY, USA). For all statistical analyses, a two‐sided level of *p *< 0.05 was considered statistically significant.

## 3 RESULTS

**FIGURE 2:**
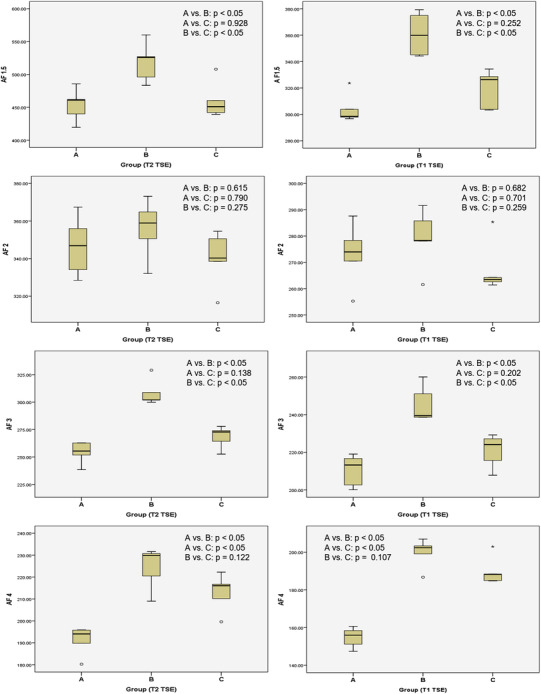
The boxplots showing comparisons of signal‐to‐noise ratio between the three groups

**FIGURE 3:**
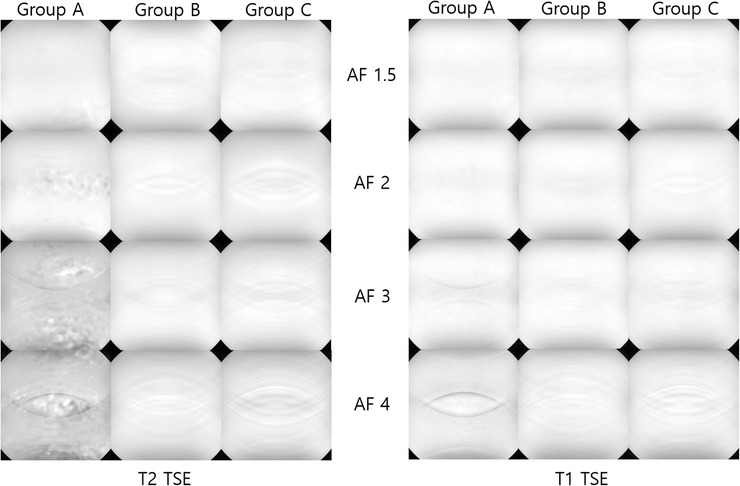
Images showing the effect of parallel imaging technique with phase oversampling distance and acceleration factors between three groups using both sequences. Group A, SENSE with phase oversampling distance of 150 mm; Group B, CS‐SENSE with phase oversampling distance of 100 mm; Group C, CS‐SENSE with phase oversampling distance of 75 mm

The measured SNR values are presented in Table 2. The SNR values had a general tendency to decrease as AF increased in all groups. The highest and lowest SNR values were shown at AF 1.5 in group B and AF 4 in group A, respectively, in both T2 and T1 TSE sequences. In T2 and T1 TSE using an AF 1.5, no statistically significant differences were found between groups A and C (*p* = 0.928 and 0.252, respectively). Moreover, no statistically significant differences were noted between groups A, B, and C in both T2 and T1 TSE sequences using AF 2 (*p* = 0.301 and 0.289, respectively; Figure 2). In comparison with AF 2 in group A, the scan time at AF 2 in groups B and C was reduced by 11.2% and 23.5% (T2 TSE) and 15.8% and 22.7% (T1 TSE), respectively, while providing comparable image quality. When using AFs 1, 3, and 4, the SNR values were significantly higher in group B than in group A in both sequences (*p* < 0.05), despite having no statistical difference at AF 2 (*p* > 0.05; Figure 3).

**TABLE 2:** Signal‐to‐noise ratios (SNRs) values for three groups according to parallel imaging technique, phase oversampling distance, and acceleration factors

**Sequence**	**AF**	**SNR**			***p*‐Value**
		**Group A**	**Group B**	**Group C**	
T2 TSE	1.5	453.63 ± 25.05	518.51 ± 29.97	460.13 ± 28.03	<0.05* ^‡^
	2	346.55 ± 15.87	355.94 ± 15.68	340.09 ± 14.83	0.301
	3	254.26 ± 10.02	308.34 ± 12.07	268.36 ± 10.09	<0.05* ^‡^
	4	191.16 ± 6.56	224.36 ± 9.68	212.96 ± 8.62	<0.05* ^†^
T1 TSE	1.5	304.14 ± 11.25	360.68 ± 16.29	319.27 ± 14.67	<0.05* ^‡^
	2	273.13 ± 11.88	279.09 ± 11.31	267.41 ± 10.08	0.289
	3	210.35 ± 8.50	245.55 ± 9.68	220.81 ± 8.92	< 0.05* ^‡^
	4	154.72 ± 5.39	199.68 ± 7.78	189.86 ± 7.51	< 0.05* ^†^

*Notes*: *p‐Values between groups A and B when statistically significant differences were indicated as per post hoc tests using the Turkey–Kramer test, †i values between group A and C, and ‡ between groups B and C when statistically significant differences were indicated as per post hoc tests using the Turkey–Kramer test. Group A, SENSE with phase oversampling distance of 150 mm; Group B, CS‐SENSE with phase oversampling distance of 100 mm; Group C, CS‐SENSE with phase oversampling distance of 75 mm. AF, acceleration factor; TSE, turbo spin‐echo.

Figure 3 shows the images used to calculate the SNR values as a function of groups, sequences, and AFs. The significant image noise and aliasing artifact were more evident at AF ≥ 2 in group A compared with those of groups B and C in T2 TSE. A reduced aliasing artifact was seen at AF ≥ 3 in group B compared with that of group A for both sequences despite the increase in aliasing artifact as the AF increased in both CS‐SENSE and SENSE.

**FIGURE 4:**
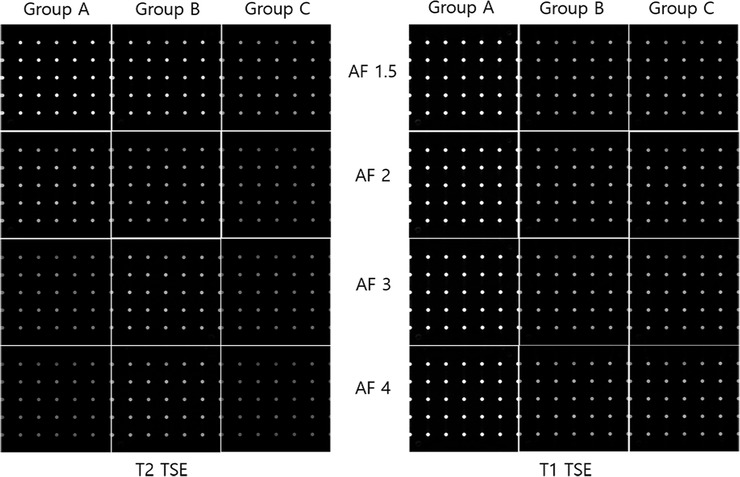
Images showing the hyper‐intense points as a function of phase oversampling distance and acceleration factors between three groups using both sequences. Group A, SENSE with phase oversampling distance of 150 mm; Group B, CS‐SENSE with phase oversampling distance of 100 mm; Group C, CS‐SENSE with phase oversampling distance of 75 mm

Table 3 presents the SSIM values obtained among the three groups using both T2 and T1 TSE sequences that were nearly identical. All SSIM values were > 0.9928 regardless of the parallel imaging technique, phase oversampling distance, and AFs. Overall, no significant differences in image quality degradation among the three groups were observed (*p* > 0.05; Figure 4).

**TABLE 3:** Structural similarity index (SSIM) values obtained from three groups according to parallel imaging techniques, phase oversampling distance, and acceleration factors

**T2 TSE**			
**Acceleration factor**	**Group A vs. B**	**Group A vs. C**	**Group B vs. C**
1.5	0.99981 ± 0.00011	0.99982 ± 0.00012	0.99993 ± 0.00001
2	0.99947 ± 0.00009	0.99978 ± 0.00009	0.99975 ± 0.00011
3	0.99932 ± 0.00016	0.99882 ± 0.00011	0.99981 ± 0.00016
4	0.99895 ± 0.00015	0.99619 ± 0.00417	0.99841 ± 0.00015

**T1 TSE**			
**Acceleration factor**	**Group A vs. B**	**Group A vs. C**	**Group B vs. C**
1.5	0.99933 ± 0.00018	0.99937 ± 0.00012	0.99993 ± 0.00001
2	0.99931 ± 0.00012	0.99932 ± 0.00012	0.99931 ± 0.00019
3	0.99714 ± 0.00016	0.99717 ± 0.00018	0.99993 ± 0.00002
4	0.99421 ± 0.00018	0.99446 ± 0.00029	0.99987 ± 0.00003

*Notes*: Their SSIM value is ∼1 when two images between groups are nearly identical. No statistical differences in SSIM values exist between groups A and B, A and C, and B and C (*p* > 0.05). TSE, turbo spin‐echo.

## 4 DISCUSSION

CS‐SENSE with a less phase oversampling distance in the current study showed a reduced image acquisition time without image quality degradation compared with that of SENSE. CS‐SENSE with 100 mm phase oversampling distance demonstrated a significantly higher SNR value without image distortion with an image acquisition time reduction by up to 17.3% for both T2 and T1 sequences compared with SENSE with phase oversampling distance of 150 mm.

The effort to reduce image acquisition time without deteriorating image quality is a crucial issue in clinical practice, and various studies related to these efforts have been conducted. Among them, CS‐SENSE has a unique undersampling method of *k*‐space by a balanced incoherent acquisition of variable density with iterative reconstruction.^6^, ^15^, ^16^, ^17^ Recent studies demonstrated that CS‐SENSE offers similar image quality to that of SENSE, with a reduction in image acquisition time.^15^, ^17^, ^18^, ^19^ These results are consistent with the current study concerning image quality and image acquisition time reduction. However, they had no explanation for phase oversampling distance and did not focus on it for their results. Thus, the result of the current study is worth providing baseline phase oversampling information for further evaluation because the current study demonstrates the effect of phase oversampling distance between SENSE and CE‐SENSE on image quality and image acquisition time. In addition, the results of the current study showed that CS‐SENSE, which has a less phase oversampling distance than that of SENSE, can reduce image acquisition time without image quality degradation. This may be explained by the differences in the undersampling method, which allows for denser sampling in the central than in the peripheral *k*‐space.^16^, ^19^ Moreover, iterative reconstruction to remove the aliasing artifact in CS‐SENSE may contribute to comparable SENSE image quality while reducing image acquisition time. Regarding SNR values and image acquisition time, CS‐SENSE was superior to SENSE with a phase oversampling distance that was 50% shorter than that of SENSE except for AF 2 as well as up to 26.4% reduction in image acquisition time. Therefore, CS‐SENSE cannot only reduce image acquisition time but also yield comparable image quality even with a shorter phase oversampling distance than in SENSE.

In contrast, both SENSE and CS‐SENSE using AF≥ 3 showed significantly increased aliasing artifacts. These results are consistent with those of other studies that reported increased noise and aliasing artifacts when using higher AFs.^13^, ^20^ Thus, more consideration should be given to the phase oversampling distance as the higher AFs are used. Given the findings of the current study, the phase oversampling distance with parallel imaging should be optimized and discussed as important to understand its influence on image quality and acquisition time.

The current study had some limitations. First, the phase oversampling distance in SENSE could not be used by setting it equal to that of CS‐SENSE. This is because SENSE can only operate over a distance of at least 60 cm including phase oversampling distance and FOV by mechanical constraints of the SENSE acquisition technique. Second, this study only used a large phantom that does not represent various organs, soft tissues, and the specific target tissues. The further study including various phantom sizes, patients, and body parts is required to demonstrate the effects of the combination phase oversampling distance with the parallel imaging technique. Finally, both T2 and T1 TSE sequences were used instead of the three‐dimensional sequence in the current experiment even though the three‐dimensional sequence has been widely used in clinical practice. Additional efforts to optimize a phase oversampling distance with either SENSE or CS‐SENSE in the three‐dimensional sequence are warranted. Nevertheless, the current study is the first study that focused on phase oversampling distance and its effects on image quality as a function of parallel imaging techniques and AFs.

## 5 CONCLUSIONS

Compared with SENSE, CS‐SENSE with a less phase oversampling distance can reduce image acquisition time without image quality degradation, despite the increase in aliasing artifact as the AF increased in both CS‐SENSE and SENSE.

## AUTHOR CONTRIBUTIONS

Guarantors of integrity of entire study, Min Hee Lee; study concepts and data acquisition, Ji Sung Jang, Ho beom Lee; data analysis and statistical analysis, Chong Hyun Suh, Ji Sung Jang, Ho beom Lee; literature research, all authors.

## CONFLICT OF INTEREST

The authors declare that there is no conflict of interest that could be perceived as prejudicing the impartiality of the research reported.
